# Getting back or giving back: understanding caregiver motivations and willingness to provide informal care

**DOI:** 10.1080/21642850.2021.1951737

**Published:** 2021-07-13

**Authors:** Mikołaj Zarzycki, Val Morrison

**Affiliations:** School of Psychology, Bangor University, Bangor, Wales, UK

**Keywords:** Informal caregiving, motivations to provide care, caregiver motives, filial obligation, willingness to provide care

## Abstract

**Background:** Informal caregivers are those providing care, which exceeds that which is typically provided, to a relative or friend with care needs. Informal caregiving constitutes the backbone of a society’s care supply and with ageing populations the need for informal care is growing. We know little as to why caregivers start caring and continue doing so, yet understanding of motivations and willingness to provide care is important if informal caregivers are to be supported. However, both motivations and willingness are inconsistently defined making it difficult to compare the empirical findings that do exist.

**Methods:** This paper reviews and synthesises thinking about the theoretical constructs of motivations to provide care and willingness to perform informal care, and presents those in relation to existing theoretical and empirical literature.

**Results and Conclusions:** Theoretical reflections based on various motivational frameworks and available empirical data are presented to illustrate that: caregiving motivations should be conceptualised as multifaceted and multiply determined; intrinsic and extrinsic motivations should not be treated as antagonistic and can occur simultaneously; the commonly applied model of extrinsic/intrinsic motivations is oversimplified and omits consideration of the diversity of caregiver motives; other motivational models can be discerned in the context of the empirical research; there are differences between motivations and willingness to provide care with the latter being more consequent to the motives; both should be considered dynamic in nature; and finally, that the two constructs may not inevitably lead to actual caregiver behaviour. The implications of these theoretical reflections for methodology and research as well as their relevance for practice and policy are indicated.

## Introduction

A high and increasing demand for care, resulting from an increasing life expectancy, lower fertility rates, a smaller family size and a growing prevalence of the elderly (Bettio & Verashchagina, 2010; Börsch-Supan, 2019; Schwarzkopf et al., 2012), make informal caregiving the backbone of a society’s care supply (Albertini, Kohli, & Vogel, 2007; Stajduhar et al., 2010). Informal caregiving can be defined as the provision of usually unpaid care to a relative or friend with a chronic illness, disability, or other long-lasting health and care needs (Revenson et al., 2016). However, the definitions used vary across studies and within official recording systems of different countries (Bauer & Sousa-Poza, 2015; Bettio & Verashchagina, 2010; Carers UK, 2019; Family Caregiver Alliance, 2015; Kemper, Komisar, & Alecxih, 2005), which makes comparisons difficult.

In many countries there is a prevalent model of family based care provision. For instance, in most Eastern European countries – considered by European Commission (Bouget, Spasova, & Vanhercke, 2016) as underdeveloped with respect to informal care support – it is common that the healthcare systems rely nearly exclusively on informal caregivers (Bettio & Verashchagina, 2010). Given the aging demographic and a societal expectation of community based, familial caregiving, there is a real risk that potential negative consequences of caregiving for the caregiver, and possibly some care recipients, will increase.

Informal care provision has been shown to come at a personal cost: providing care for a loved one who is dealing with illness or disability can be stressful, time-consuming, physically exhausting and this can negatively affect the process and experience of caregiving and the physical and psychological outcomes of both giver and receiver. Previous studies, including several reviews (Angelo & Egan, 2013; Chiao, Wu, & Hsiao, 2015; Faronbi, Faronbi, Ayamolowo, & Olaogun, 2019; Faucher & Garner, 2015; Lu, Mårtensson, Zhao, & Johansson, 2019; Parveen, Morrison, & Robinson, 2011; Viitanen, Winblad, Tuomilehto, Rovio, & Ka, 2007; Williams, Morrison, & Robinson, 2014), have typically highlighted prevalent negative consequences with regards to caregiver burden and strain, unmet needs and concerns. Care recipients may also report feelings of being worthless, lonely, fearful and not in control of decision making with regards to their care receiving experience (Clissett, Porock, Harwood, & Gladman, 2013; Cowdell, 2010; Stenwall, Jönhagen, Sandberg, & Fagerberg, 2008). Caregiving gains are present also but less often reported, including for example personal satisfaction, growth, improved relationship with the care recipient, gaining spiritual/religious blessings or learning new skills (Faucher & Garner, 2015; Murphy, 2005; Parveen et al., 2011; Quinn & Toms, 2019; Williams et al., 2014; Yu, Cheng, & Wang, 2018).

Although we have limited knowledge of the influence of personal motivations and willingness to care on caregiver experience and outcomes, it has been shown that caregiving motivations and willingness play a vital role (Lyonette & Yardley, 2003; Morrison & Williams, 2020; Parveen, Morrison, & Robinson, 2013; Quinn, Clare, & Woods, 2010). We also have limited understanding of the factors that underly, promote and maintain motivations and willingness to provide informal care (Burridge, Winch, & Clavarino, 2007; Parveen et al., 2011; Parveen & Morrison, 2012) in the first place. If informal care is to be sustained for care recipients across the globe, it is important to identify factors that ameliorate negative caregiver experience and outcomes, otherwise it is likely that any initial motivations and willingness to provide informal care will subside amongst informal carers.

It is difficult to compare existing empirical findings due to vague and conflicting definitions of motivations and willingness to provide care, and of informal caregiving itself. The lack of clear definition and operationalisation of such concepts is a major lacuna in the field. This paper aims to review and synthesise thinking about the theoretical constructs of motivations to provide care and willingness to perform informal care, and present those in relation to existing theoretical and empirical literature. Firstly, the construct of caregiving motivations is investigated, then that of willingness to provide care with further elaboration on the relationships, similarities and differences between the two constructs. There is a need for empirical research to address personal, societal and cultural influences on motivations and willingness to provide care. However, it would be ill-advised to turn practical attention toward these caregiving constructs by means of tailored interventions until a more robust and reliable synthesis of empirical evidence is achieved and more consistent conceptualisation has been achieved.

## Motivations to provide care

The concept of motivation is central to many psychological studies of behaviour, including studies of health care systems, academic performance and personal health and well-being (Cerasoli, Nicklin, & Ford, 2014; Fisher, Fisher, & Harman, 2003; Franco, Bennett, & Kafner, 2002; Hidi & Harackiewicz, 2000; Ryan & Deci, 2000). There is no standard, unified definition for motivation as it is conceptualised differently depending on the underlying theoretical stance taken. In broad terms, motivation is a ‘drive’ to act – often discerned biologically, for example pertaining to thirst or hunger motives, but also psychologically with reference to anticipated social or personal outcomes. Therefore, individual motivation can be understood to provide the explanation/reason for how/why individuals react and fulfil their (commonly biological) needs, i.e. the route leading to one’s behaviour or as the psychological construct that triggers someone’s need or desire to replicate behaviour (Cook & Artino, 2016; Maslow, 1943). Motivation is described as the process responsible for initiating, guiding and maintaining goal-oriented behaviours (Hidi & Harackiewicz, 2000; Ryan & Deci, 2000). Motivation drives human beings to take action to achieve a goal or to fulfil a need or expectation (Ryan & Deci, 2000). Other than that, there are a few other definitions that depict motivations as: an internal condition that arouses, directs and maintains behaviour (Woolfolk, 2013); the reason underlying certain behaviours (Collins & Jones, 1997; Powers & Whitlatch, 2016; Statham, 2003); an attribute that instigates energy, direction, the reason for our behaviour and ‘what’ and ‘why’ people do something (Ryan & Deci, 2000). All three conceptualisations similarly indicate that motivations underpin conscious actions, even if the motivations themselves (whether defined as a ‘condition’, ‘reason’ or ‘attribute’) may be unconscious, subconscious or conscious.

### Theoretical frameworks for caregiver motivations

Theoretical frameworks within which caregiver motivations have been considered include those of altruism, empathy, egoism, responsibility: *Empathy Induced Altruism Hypothesis* (Bateson, 1991) proposes that motivation may be produced by empathic emotion and thus empathetic reactions of the carers; the *Self-Interest Model* (Greenberg, 1990; Lind & Tyler, 1988) claims that a caregiver is concerned with their own situational outcomes in adopting the caregiving role*;* the concept of reciprocity is the basis for the Social Exchange Theory and its two derivative forms: the (In)equity Theory and the Theory of Indebtedness; *Social Exchange Theory* (Adams, Berkowitz, & Hatfield, 1976; Homans, 1961) highlights the issue of balance whereby carer and care recipient gauge a relationship and the input and output regarding relationships, cooperation, and competition in a wider caregiving context (e.g. when more than one person is engaged in the caregiving); the *Indebtedness Theory* (Green, Greenberg, & Willis, 1980), based on which people provide informal care because they want to reduce the feeling of indebtedness that they have toward the care recipient (with the returning behaviour reducing the indebtedness as a form of social exchange); the *(In)equity Theory* (Adams, 1965) in which caregivers take care of their care recipients to achieve ‘equity’ of inputs expected in their relationship whereby they might give up caregiving if they have a feeling of imbalance and a sense ‘unfairness’ or ‘inequity’; *Tit for Tat Theory* (Frank, 2002) deems the caregiving behaviour as mutually beneficial for the carer and care recipient*;* the *Reciprocal Altruism Model* (Barber, 2010) offers a biological theory stating that caregiving acts are ‘reciprocally altruistic’ as favoured by natural selection because in the long run they may benefit the caregiver; *Kin Selection Model* (Humphrey, 1997) involves helping relatives for the survival and reproduction of the individual and closest kin*;* the *Normative Approach* (Homans, 1961) offers a general attempt to describe caregiver motivations in terms of being guided by societal norms; *Commitment Theory* (Blieszner & Shifflet, 1989; Johnson, Caughlin, & Huston, 1999) recognises internal and external factors, influencing carer commitment to helping a care recipient, especially with a recognition of psychological factors contributing to commitment; *Self-determination Theory* (Ryan & Deci, 2000) distinguishes intrinsic and extrinsic motivations through the examination of innate psychological needs. As can be seen across these several theories, some of them embrace motivations to provide care by pointing to the caregiver ‘getting something back’ (Adams et al., 1976; Barber, 2010; Frank, 2002; Greenberg, 1990; Homans, 1961; Humphrey, 1997; Lind & Tyler, 1988), whereas the others put more emphasis on the carer ‘giving something back’ (Adams, 1965; Adams et al., 1976; Bateson, 1991; Green et al., 1980; Homans, 1961). Table 1 outlines the theories encompassing caregiving motivations with the accompanying summary descriptions.

**Table 1.** Theoretical frameworks for informal caregiver motivations.

Theory	Author	Main focus
*Empathy Induced Altruism Hypothesis*	Bateson (1991)	Motivation produced by empathic emotion and thus empathetic reactions of the carers
*Self-Interest Model*	Greenberg (1990); Lind and Tyler (1988)	A caregiver concerned with their own situational outcomes in adopting the caregiving role
*Social Exchange Theory*	Adams et al. (1976); Homans (1961)	Motivation based on the issue of balance whereby carer and care recipient gauge a relationship and the input and output regarding relationships, cooperation, and competition in a wider caregiving context
*Indebtedness Theory*	Green et al. (1980)	Informal care as a returning behaviour reducing the feeling of indebtedness toward the care recipient
*(In)equity Theory*	Adams (1965)	Caregivers provide informal care to achieve ‘equity’ of inputs expected in the relationship and they might relinquish the caring responsibility if experiencing a sense of ‘inequity’
*Tit for Tat Theory*	Frank (2002)	Caregiving behaviour as mutually beneficial for the carer and care recipient
*Reciprocal Altruism Model*	Barber (2010)	Biological theory positioning caregiving ‘reciprocally altruistic’ acts as favoured by natural selection because in the long run they may benefit the caregiver
*Kin Selection Model*	Humphrey (1997)	Helping relatives for the survival and reproduction of the individual and closest kin
*Normative Approach*	Homans (1961)	Caregiver motivations are informed by societal norms
*Commitment Theory*	Blieszner and Shifflet (1989); Johnson et al. (1999)	Internal and external factors (e.g. psychological), influencing carer commitment to helping a care recipient
*Self-determination Theory*	Ryan and Deci (2000)	Intrinsic and extrinsic motivations based on the examination of innate psychological needs

Motivation has also been classified as consisting of one of six or seven types on a continuum from external (controlled) to internal (autonomous) contingency, nested hierarchically by global, contextual, and situational level specific factors (Vallerand, 1997). Ryan and Deci (2000) and Vallerand (1997) have provided the grounds for the intrinsic and extrinsic typology of motivation which is seen more commonly within the empirical literature around informal care (Cameron & Pierce, 1994; Lyonette & Yardley, 2003; Ryan & Deci, 2000). In this literature a dualistic theory, i.e. intrinsic versus extrinsic, is almost exclusively applied (Feeney & Collins, 2003; Kim, Carver, & Cannady, 2015; Lyonette & Yardley, 2003; Quinn et al., 2010; Quinn, Clare, McGuinness, & Woods, 2012; Walker et al., 2019). We will argue that researchers should take a closer look at the conceptualisation of these notions in informal caregiving research and consider evidence of more complex caregiving motivations (i.e. that they are multiply faceted and are not necessarily mutually exclusive) as this will enable such factors to be assessed more robustly and appropriately.

### Initiation and continuation motives

We propose to differentiate between initiation and continuation motives. Whilst the first ones are concerned with the reason(s) why a person decided to take on the caregiving role when such a need has arisen, the second ones pertain to motivations for continuing to care when already providing informal care or considering it again in the future. The caregiving journey can be conceptualised as consisting of stages, for example, stages of preparation, acquisition, enactment and role disengagement (Aneshensel, Pearlin, Mullan, Zarit, & Whitlatch, 1995; Lawton et al., 2000). The limited, mainly qualitative, data suggests that different motivations may be apparent at different stages of caregiving (depending on the illness type, relationship quality, etc.). For example, it has been proposed (Schulz, Biegel, Morycz, & Visintainer, 1989) that in the early stages carers could be motivated by altruistic motives whereas in the later stages carers may be more egotistically motivated. Hsu and Shyu (2003) in their study exploring the social exchanges of informal caregivers in an Asian context described changes in motivations starting with reciprocity motives, going through religiosity and expectation of reciprocity in the future, and ending at perceived social pressure when caregiving demands were higher. In other qualitative studies carers described past or expected shifts in motivations from love and a sense of responsibility to seeking relief from the obligations and burden of care (Browne Sehy, 1998; Foster, 2012). In another study, carers stated that their role became easier over time due to the care duties becoming habitual (Parveen et al., 2011). However, in a qualitative systematic review of determinants of motivations in dementia informal caregiving (Greenwood & Smith, 2019) it proved difficult to separate out descriptions of initiation motives to care as compared to motivations for continuing to care. These authors concluded that a high proportion of continuation motives appeared similar to initiation motives for caring, for example: love towards a care recipient, perceived obligation to care, avoiding paying or distrust of nursing homes, the belief that carers could provide higher care quality compared to professionals, companionship and satisfaction derived from the role, the belief that no one else was available (Greenwood & Smith, 2019).

The effects of time, or of transitions in the role on caregiver outcomes, have been explored quite extensively leading to empirical verification of two opposing theoretical proposals of ‘wear and tear’ and of ‘recovery’ (a short summary of evidence for both is presented by Brown & Bond, 2016). Whilst it is evidenced that transitions can affect caregiver outcomes, especially in relation to the impact of cessation of caregiving on carer well-being, transitions in the role and time spent in the role may also impact motivations to care. This requires further empirical testing as suggested by qualitative literature (Browne Sehy, 1998; Foster, 2012; Hsu & Shyu, 2003; Morrison & Williams, 2020; Parveen et al., 2011). The suggested adaptation to the role, making it more habitual as well as unexpected rewards and reinforcers, especially with extrinsic motivators emerging over time (e.g. implementation of formal services, carer benefits) possibly play an essential role in shaping continuation motivations. In a rare longitudinal qualitative study (Morrison & Williams, 2020) one caregiver expressed having found freedom through employing formal services and having relinquished a primary desire to be the sole carer – regaining some independence constituted a new motivation to continue the role. There are a few other qualitative studies indicating how respite care services (Russell, 2001; Sterritt & Pokorny, 1998; Tretteteig, Vatne, & Rokstad, 2017; Tretteteig, Vatne, Rokstad, & Rokstad, 2017), home support services (Gerdner, Tripp-Reimer, & Simpson, 2007; Lewis, Curtis, & Saucier Lundy, 1995; McDonnell & Ryan, 2014; Öhman & Söderberg, 2004) and other formal services (Lee, Lee, & Lee, 2019; Neufeld & Harrison, 1998; Stajduhar, Martin, Barwich, & Fyles, 2008) play an important role in shaping continuation motivations. Interestingly, monetary incentive (in terms of explicit benefits or inheritance related factors) explored in both a qualitative (Kietzman, Benjamin, & Matthias, 2013) and quantitative (Caputo, 2002) studies turned out to be of secondary importance, which – according to the authors of the studies – may suggest the motivational primacy of adherence to social norms about caregiving (e.g. filial responsibility). However, social desirability may also play a role in the self-reported motivations for caring, with monetary motives perhaps considered socially undesirable and therefore underreported (e.g. in comparison to social expectations around familial caregiving as expression of love and obligation toward the care recipient). As caregivers usually express more than one caregiving motivation, the understanding of the importance/salience of each expressed motive comprises a complex issue. Future research should take into account the differentiation between initiation and continuation motives as well as apply more complex understanding of them in order to further understand carer support needs.

Within healthcare research the concept of clinical inertia, i.e. the recognition of the medical problem between a patient and a physician, but with failure to act upon this (Phillips et al., 2001; Valencia, Florez, & Palacio, 2019), has been shown to have negative health consequences (e.g. delayed treatment, Valencia et al., 2019). It may be that a similar concept around inertia in caregiving may exist that prevents or delays caregivers in seeking necessary support when motivations falter or when care demands change.

### Model of extrinsic versus intrinsic caregiving motivations

Overt and latent motivations for providing informal care have been shown to be crucial in understanding the extent to which caregiving experience influences caregiver and care recipient outcomes (Feeney & Collins, 2003; Kim et al., 2015; Lyonette & Yardley, 2003; Quinn et al., 2010, 2012; Revenson et al., 2016; Walker et al., 2019). The consideration of motivation as either extrinsic or intrinsic (Cerasoli et al., 2014; Ryan & Deci, 2000) as commonly employed in the field is a simplification of the existing motivational theory but perhaps for very practical reasons a measure was developed based on that distinction – ‘Motivations in Elder Care Scale’ (MECS; Lyonette & Yardley, 2003). This pragmatic approach has yielded important findings (Lyonette & Yardley, 2003; Quinn et al., 2010), however motivational regulation is a complex psychological construct. Whilst the extrinsic-intrinsic distinction has proven popular in the caregiving context (Feeney & Collins, 2003; Kim et al., 2015; Lyonette & Yardley, 2003; Quinn et al., 2010, 2012; Walker et al., 2019), it takes a highly generalised and simplified approach when compared with more intricate motivational models such as the *Self-determination Theory* by Ryan and Deci (2000) or hierarchical model of intrinsic and extrinsic motivation by Vallerand (1997) – for the summary of theories see Table 1. The discussion of what is missed by this simplification is presented below.

Extrinsically motivated behaviours are governed by the expectancy of instrumental consequence, i.e. gain and loss (incentives such as the attainment of a reward, avoidance of a punishment or the achievement of a valued outcome; Porter & Lawler, 1968; Vroom, 1964). Intrinsic motivation refers to people’s spontaneous tendency for the engagement in the behaviour for its very own sake (behaviour is itself purposive), seeking out challenges and exercising skills and knowledge; a behaviour not being instrumental toward some other outcome, *even* in the absence of operationally separable rewards (Di Domenico & Ryan, 2017; Ryan & Deci, 2000). Grounded in this distinction, motivations to provide care are usually considered to emerge from internal influences (intrinsic motivations) or from external influences (extrinsic motivations). Intrinsic motives refer to emotional bonding, feelings of usefulness and perception of personal choice in the decision to provide care, while extrinsic motives are related more to social expectations, a sense of obligation (e.g. filial obligation) and perception of the lack of choice in caregiving decision (Revenson et al., 2016).

Meta-analyses examining the impact of incentives and intrinsic motivation on performance have drawn inconsistent conclusions (Cameron & Pierce, 1994; Deci, Koestner, & Ryan, 2001), likely due to the fact that the operationalisations of intrinsic motivation have drawn from different theories and the notion itself remains problematic (Cerasoli et al., 2014). For instance, can we still consider a previously reported intrinsic motivation to be valid at the point when a person starts to consider any kind of incentive or reward for their action? What is the role of extrinsic incentives in maintaining intrinsic motivation? Most of the incentives described as extrinsic motivations are quite clearly external gains or losses (e.g. welfare benefits or monetary awards, health benefits, praise, recognition), but what about other outcome expectancies relevant for personal experience (e.g. satisfaction and enjoyment from sustaining a good relationship)? Are these expectancies comparable to incentives but internalized? Therefore, is intrinsic motivation undermined by extrinsic rewards – the so-called undermining effect (see Deci et al., 2001)? The operationalisation of intrinsic motivation remains problematic and is typically defined in contrast to the extrinsic motivation.

### Defying the boundary of intrinsic–extrinsic distinction

As intrinsic motivation is conceptualised as a notion contrary to external motivation, this implies an exact boundary between the two motive forms. We can distinguish at least between two views on intrinsic motivation. The first considers intrinsic motivation as the ‘supreme authentic force of behaviour regulation’ (separate from incentives motivation) and is presented in Frankl’s (2011) existential theory. In this theory the fulfilment of values provides a person with a meaning that constitutes a core mechanism of intrinsic motivation (based on noetic tension). From this perspective intrinsic motivation is not an epiphenomenon resulting from individual aspirations or expectations but is implicitly involved in discovering and encompassing meaning in an ongoing way through values realisation and/or their fulfilment (Frankl, 2011; Maslow, 1943). The second perspective of intrinsic motivation is based on the feelings of satisfaction or enjoyment derived from the action (Di Domenico & Ryan, 2017; Ryan & Deci, 2000) and implies that outcome expectancies may play a vital role in this type of motivation (e.g. engagement in caregiving to maintain satisfaction in a relationship between the carer and the care recipient). However, it is empirically unknown whether and to what degree (different) incentives moderate the predictive validity of intrinsic motivation (Cerasoli et al., 2014). It is important to note that the emphasis on an exact boundary between extrinsic and intrinsic motivations may result in findings overlooking their interlinked nature within the motivational process. We could also argue further that every extrinsic motivation is not completely external as it is accompanied by some internal emotions and some inner value judgement (e.g. caregiving is a ‘good and right’ thing to do) or decision (e.g. a caregiver providing care initially only on the condition that financial benefits are received may subsequently derive satisfaction and enjoyment from the role and seek a strengthened relationship with the recipient). Therefore, it might not be the emotional component nor the incentive/expectation or aspiration that best differentiates between the two motivational types but rather the reason underlying the decision to initially undertake the task. Motivations, as suggested before, cannot be considered outside the temporal aspects of caregiving, i.e. the caregiving journey and indeed that of the care recipients’ illness and care needs bring potential for changing caregiving motivations (initiation and continuation motives). Moreover, Cerasoli et al. (2014) propose that incentives and intrinsic motivations are not antagonistic and are best considered simultaneously. This remark is significant when considering complex caregiver motives in that *both* intrinsic and extrinsic motivations may be functional in a caregiver context. It is, we consider, justifiable to consider an interactive juxtaposition of extrinsic and intrinsic motivations on caregiver outcomes. Relating to the previously mentioned distinction noticeable in theories of caregiving motivations between ‘getting back or giving back’, we would argue that both can happen.

As motivation is multifaceted (Kanfer, Chen, & Pritchard, 2008) and multiply determined (Baker, Jensen, & Murphy, 1988), factors contributing to the influence *on* motivations should be considered separately to the influence *of* motivations on caregiver outcomes. A caregiver may be motivated to provide care for many reasons, by feelings of duty and responsibility as well as of guilt. Doty (1986) identified three factors underlying family caregiving motivations: love and affection, desire to reciprocate past help and societal norms. Schulz et al. (1989) present their list of motives including: altruism, egotistic motives, social norms. Some authors suggest that emotions play a significant role in revealing basic latent motives (Hermans, 1999; Hermans-Jansen, van Gilst, Hermans, Hermans-Jansen, & van Gilst, 1987) underlying the manifest level of behaviour regulation. Caregivers’ motivations can be affected by past reinforcement of helping behaviour (Feeney & Collins, 2003) or if helping previously had negative consequences then the motivation may be not to help. Equally, the lack of perceived or actual resources or skills to provide care is the next factor contributing to motivations to provide care. Characteristics of the care recipient and their relationship with a carer have also their contribution, i.e. motivations depend on personal features of the caregiver and the recipient (e.g. attachment style, self-esteem, gender) and perceptions of the relationship (Feeney & Collins, 2003). Corey and McCurry (2017) claim that the main reason underlying the initiation of the caregiver role comprises the subjective values placed upon caregiving which are biased/influenced by societal values and expectations. These subjective values exert a sense of obligation upon the individual (Corey & McCurry, 2017) which is said to be the primary caregiving motivation. They propose a theoretical division between *authentic* and *inauthentic caregiving motives*, a different dualistic conception compared to extrinsic versus intrinsic motivations. Inauthentic motives include guilt, sense of duty, powerlessness, shame and social conformism, whereas authentic motives are described as pursuing an action for its own sake dependant on the individual cause and choice made. Value and meaning are inherent in this as the main source of motivations.

As suggested by Quinn et al. (2010) the grouping of motivations under a theoretical framework may be more useful than the conceptualisations seen in existing caregiving studies (e.g. Carruth, 1996; Kabitsi & Powers, 2002; Lee & Sung, 1997). Kohli and Künemund (2003) describe a model in which the motives for help-giving among next of kin have been considered on two dimensions, i.e. *altruism – sense of duty* and *direct exchange – indirect reciprocity*. Altruism regards the caregiver concern for the well-being of the care recipient, whereas a sense of duty comprises an internalised normative obligation. Direct exchange is about the caregiver interest in getting something in return immediately and indirect (delayed) reciprocity concerns returning what one has received earlier, passing it on to the next generation. It is also worth noting that another approach identified dimensions of expectations – personal or particularistic, and normative or universalistic (Ganong & Coleman, 2005), with the former two referring to parents’ particularistic expectations of their own children and the latter two pertaining to norms that regulate obligations between caregiving children and their parents.

The identified moderators and dimensions of motivations to care employed in research will affect the extent to which we can understand their effect on carer behaviour. Avoiding a comparison of whether external incentives or intrinsic motivation is the ‘better’ or more ‘beneficial’ driver of caregiving should be the goal. From a dimensional point of view a caregiver may provide care because they deem it is their obligation (e.g. for social recognition consequent to meeting a perceived social expectancy – extrinsic motivation) *and* also because they feel good in the role and experience the fulfilment and satisfaction while engaged in caregiving (intrinsic motivation). Extrinsic and intrinsic motives may not be mutually exclusive. General experimental research in the field of motivation suggests that contingency and the kind of task/performance/behaviour may constitute important moderators of motivations overall. Four contingency categories tested in lab research by Deci et al. (2001) include whether an incentive was promised for engagement in the task, for completion of the task, depending on the level of task performance, or whether the incentive was non-contingent on the task action. All incentives were hypothesised to reduce intrinsic motivation (through providing an ‘undermining effect’). A meta-analysis focused on the examination of relationships between motivation and performance supported the perspective that controlling incentives (e.g. a caregiver providing care due to legal obligation) reduced intrinsic motivation but supporting incentives (e.g. a caregiver providing care because it strengthens their relationship with a care recipient) enhanced intrinsic motivation (Cerasoli et al., 2014). A more proficient operationalisation of caregiving motivations is needed, i.e. including their multifaceted, fluctuating nature and considering juxtaposition of intrinsic and extrinsic motivations, in order to understand the effect motivational moderators may have on motivations for caring.

Furthermore, it might need to be shown empirically not only *if* but also *when* and *why* intrinsic motivation and incentives work together to impact the behaviour. From one of the perspectives of intrinsic motivation theories (Deci et al., 2001; Deci, Koestner, & Ryan, 1999), incentives are irrelevant in terms of initial motivation but not when it comes to the outcome experience. We can hypothesise that people who are motivated intrinsically, not by incentives (which may comprise mere epiphenomena to the motivated action itself) have better caregiver outcomes than those motivated *only* extrinsically (Cameron & Pierce, 1994; Lyonette & Yardley, 2003). However, this has not been tested empirically and whether incentives increase intrinsic motivation by acting as a form of positive reinforcement remains unclear.

In concluding this section we would note that in terms of studies of caregiving motivations, research has primarily been based on cross-sectional studies although several have noted that different types of motivations may be present at different times during the ‘caregiving career’ (Browne Sehy, 1998; Foster, 2012; Hsu & Shyu, 2003; Morrison & Williams, 2020; Parveen et al., 2011; Quinn et al., 2010; Schulz et al., 1989). Further longitudinal research is necessary to strengthen support for the points made herein. In addition, there is a field of work that addresses the notion of willingness to provide care which may usefully add to considerations of caregiver motivations. In the next section we consider how this concept has been described and examined, and whether it bears any relations to our understanding of caregiving motivations.

## Willingness to provide care

### Relationships between willingness and motivations to provide care

Willingness to provide care has been defined as part of a caregiver’s *attitude* towards providing support for an individual, whether the support required is a current or future need (Abell, 2001). The notion of willingness to provide care has been presented independently from the motivations to care in quantitative literature (e.g. Abell, 2001; Lyonette & Yardley, 2003), although we will argue here that the former may be understood also as a consequence of the underlying motivations to provide care with which it interacts. Both motivations and willingness to care have been considered as synonymous concepts in qualitative research without differentiating between the two (Cash, Hodgkin, & Warburton, 2013; Holroyd, 2001; McDonnell & Ryan, 2014; Mok, Chan, Chan, & Yeung, 2003; Williams et al., 2014), e.g. distrust of formal care may constitute the reason why a person has both decided to provide care themselves (which pertains to a motivation or reason for the caregiving behaviour) and at the same time distrust of formal caring institutions may influence willingness to care. The differences between motivations and willingness to provide care are summarised in Table 2 and we argue that there is a connection between the two notions. Simply stated, i.e. without holding to account different dimensions of motivations to care, we can hypothesise that an intrinsically motivated person might be more *willing* to care whereas an extrinsically motivated carer may feel more *obligated* to provide care and therefore be less willing to do so. Similarly, it can be hypothesised based on a bidirectional influence that personal experience/outcomes of willingness to care (in different tasks) may in turn modify motivations to provide future care. For instance, carers may be strongly motivated to provide care because they feel love and affection toward the care recipient, but simultaneously or subsequently they might find themselves unwilling to fulfil some caring tasks (e.g. certain nursing tasks) and this may cause feelings of guilt that interfere with the basic motivation, i.e. a shift from intrinsic to extrinsic motives. As care tasks are varied, it cannot be assumed that motivations will inform willingness equivalently across all these tasks. Furthermore, we recognise that a dichotomy of caregiving motivations (intrinsic, extrinsic) seen in the aforementioned examples can be challenged.

**Table 2.** Differences between motivations to provide care and willingness to perform caregiving tasks.

	Motivations to provide care	Willingness to perform informal care
Definition	Caregiver’s *orientation* and *level* of motivation concerning their underlying goals, attitudes, beliefs and values that give rise to providing care for an individual in need	Caregiver’s *attitude* towards providing support for an individual, whether the support required is a current or future need (Abell, 2001)
Main focus	The why of action (why does someone provide care?); the reasons why a person engages in a particular behaviour	The ‘what’ of action, i.e. anticipated/intended or actual responses to the ill person’s current or future needs; the extent to which a carer would/intends to perform diverse caregiving tasks
Exemplary variation types	Intrinsic/extrinsic; altruistic/egoistic; autonomous, introjected, external	Caregiving tasks: emotional, nursing and instrumental
Examples	‘I provide care because it’s something I deeply value doing’; ‘I provide care because I would feel guilty if I didn't’	‘I’m completely willing to do someone’s laundry’; ‘I’m somewhat unwilling to comfort someone who is upset’

Willingness to perform caregiving tasks, as suggested by the name of the construct, is more behaviourally conceptualised, i.e. it is generally construed in relation to a behaviour. Similarly, communal motivation – a broader concept than willingness to provide personalised informal care – is actually akin to conceptualisation of willingness as it is defined as generally being motivated to *show care and concern* (i.e. a manifest behaviour) for the welfare of others (Clark & Mills, 2011; Le, Impett, Lemay, Muise, & Tskhay, 2018). Whilst assessed outside the informal caregiving context, there are two core interests of this concept, i.e. one concerns how willing people are to care for other people’s needs; the other one pertains to the reciprocal expectations of this willingness from the other. Since the first is similar to the conceptualisation of willingness in terms of the costs the person would be willing to incur to benefit the other (Abell, 2001; McDonell, Abell, & Miller, 1991), communal motivation can serve as a proxy concept of willingness to provide care (Abell, 2001; Mills, Clark, Ford, & Johnson, 2004). The developed Measurement of Partner-Specific Communal Motivation (Mills et al., 2004) can also be used to assess willingness to provide care generally, allowing insight into the emotional and reciprocal aspects of the notion (e.g. in terms of the distress or guilt a carer would feel if they were unable to meet the care recipient’s needs). However, this measure does not include the assessment of ability and tasks specification which, as discussed later, remains crucial to the operationalisation of the notion as stated by Abell (2001).

### Towards the understanding of willingness to provide care as a process

As with motivations to care discussed previously, two different perspectives of willingness to care can be derived from the literature concerning the construct’s stability and temporal orientation. In its definition an attitude towards providing care can be considered as a stable, permanent concept (Wilson & Hodges, 1992) or as a dynamic and fluctuating notion, *attitudes-as-constructions perspective* (Schwarz & Bohner, 2001)*.* Studies using cross-sectional designs encapsulate willingness to provide care in the context of sustaining a relationship over time and consider it as a general and relatively permanent attitude (Abell, 2001; Wells & Over, 1994). However, willingness to care examined at the trait level overlooks its variability – as stated by Pearlin (1994, p. 18): ‘[caregiver] outcomes are best thought of not as end states but as patterns of continuity and change that parallel continuities and changes in the conditions of caregiving’. Temporal orientation encompasses whether willingness to care refers to the actual current situation or to a future need of support, i.e. if willingness to care is assessed before or after a caregiver takes on the caregiving role. At the current time research is lacking in prospective longitudinal evidence, thus limiting a thorough review of the temporal aspects of the notion. However, qualitative longitudinal case studies reported by Morrison and Williams (2020) identified shifts in willingness over time dependent on the care recipients condition (i.e. deterioration) and the likely types of care tasks that were anticipated. As with motivations to provide care, it is crucial that initial willingness be differentiated from continuation willingness based not only on the tasks performed but also factors discerned prospectively in the caregiving journey (e.g. stage and severity of care recipient’s illness, caregiver’s life stage, family structure, geographical proximity, financial means to provide care, caregiver employment commitments).

Willingness to provide care has also been operationalised to distinguish between three different caregiving tasks, i.e. emotional, physical and instrumental tasks (Abell, 2001; McDonell et al., 1991). The Willingness to Care Scale by Abell (2001) reflects two distinct and related components when measuring the concept: *ability* (representation of the tasks carers *believe they could* perform if necessary) and *willingness* (representation of the tasks carers *would* perform). The distinction between ability and willingness to provide care is crucial as it cautions against making assumptions about behaviour consequent to a caregiver’s perceived willingness. As suggested by Seddon and Robinson (2015), this is important when translating research findings into practice – motivations and willingness for caring are often taken for granted once the ability to care has been confirmed, but this may not be the case.

Moreover, the existing definition of willingness to provide care supports a distinction between the *actual willingness* and *hypothetical willingness* to provide care in the future. The first refers to the situation when support that is provided addresses a current need, whereas the latter pertains to anticipatory willingness, providing support if such a need arises (i.e. if a person becomes a caregiver). Studies have addressed both forms, for example, of willingness to care in actual caregivers (e.g. Parveen, Morrison, & Robinson, 2014) or of willingness to care amongst people who have not yet been caregivers but with an assumption that they will likely care for a family member in the future (e.g. Goldberg-Looney, Perrin, Morlett-Paredes, & Mickens, 2017). A further related notion is that of *willingness to care again*, which refers to past caregivers, i.e. carers who transition out of the role as a result of the death or institutionalisation of their former care recipient. In this case the emphasis is put on how willing the carers would be to provide care again under similar future circumstances (e.g. Johnson et al., 2016).

### Determinants of willingness to provide care

In seeking understanding of differences in willingness to care seen in the literature several factors have been identified. For example, McDonell et al. (1991) theorised that caregiver resources and perceived filial obligation (which could be considered as motivations to provide care) influence willingness to provide informal care within the family context. Burridge et al. (2007) in their systematic review of 17 studies identified 4 clusters of willingness to care indicators (demographic, physical, psychological and social). These can be seen in (mainly cross-sectional) studies that have examined potential predictors of willingness to provide care in the future. These include basic demographic characteristics (e.g. gender, age, place of residence, civil status, etc.), family structure (number of brothers and number of sisters), family dynamics (e.g. attachment, communication style), religious affiliation (none vs. Christian and others), masculinity/femininity, functional impairment of a care recipient (Burridge et al., 2007; Dykstra & Fokkema, 2012; Lieberman & Fisher, 1999; Wells & Johnson, 2001) and the care recipient’s illness characteristics (Williams et al., 2014). For instance, caregiver willingness decrease with older age, but not for all; low income may also negatively influence willingness to provide care (Burridge et al., 2007). Care recipient’s illness characteristics pertaining to the predictability of an illness, its duration, the nature and intensity of change can influence willingness to provide informal care (Burridge et al., 2007; Wells & Over, 1994; Williams et al., 2014), however, this still remains understudied. In one large study conducted in the Netherlands people with higher education reported lower willingness to care compared to people with lower levels of education (Dykstra & Fokkema, 2012), conversely to an earlier and smaller study conducted by Lieberman and Fisher (1999) in the U.S.A. People committed to their religious communities and regularly attending religious services were also found to be more willing to provide care (Dykstra & Fokkema, 2012), with influences of ethnicity seen in that Muslim respondents and migrants coming from more collectivist cultures expressed the highest willingness to provide care compared to those of Dutch descent (Dykstra & Fokkema, 2012). In addition, contrary to expectations lower familial willingness to provide care occurred when the care recipient lived with their spouse than when they lived alone or with offspring (Lieberman & Fisher, 1999). Moreover, willingness to provide informal care was higher among adult children whose parents remained married than those whose parents had divorced or separated; which led also to the consideration of the role attachment played in willingness to provide care (Wells & Johnson, 2001). Gender differences in willingness to provide care seem to depend heavily on the caregiving motivations and caregiving tasks involved, e.g. male carers preferred traditionally male responsibilities such as managing finances, over traditionally female responsibilities such as personal care (considered a ‘women’s work’) (e.g. Campbell & Martin-Matthews, 2000, 2003).

Qualitative empirical literature does not distinguish between caregiving motivations and willingness to provide care unlike a small body of quantitative evidence in which both constructs co-exist separately and where limited links between them have been examined. Keefe, Rosenthal, and Beland (2000) found that caregiving motivation such as filial obligation was a strong positive predictor of willingness to provide care. Similarly, higher filial obligation was related to greater willingness to provide gender-neutral care (e.g. transportation, helping with shopping) and traditionally female care (e.g. domestic assistance, personal care) with higher income men providing significantly less traditionally female care than lower income men (Campbell & Martin-Matthews, 2003). Chappell, Funk, Chappell, and Funk (2011) compared Caucasian and Chinese caregivers in their willingness to provide care and found that even though Caucasians reported lower norms of filial responsibility, they were nevertheless willing to provide extensive care on the level similar to this reported by Chinese carers. Horwitz's (1993) finding that carers with higher levels of obligation were not more willing to care contradicts the findings seen in the other three studies (Campbell & Martin-Matthews, 2003; Chappell et al., 2011; Keefe et al., 2000), but the author included a further measure of motivations, that of reciprocity, with findings demonstrating that reciprocity could be predictive of willingness to provide care. The limited empirical evidence presented is insufficient to draw any conclusions on the relationship between motivations and willingness, i.e. how motives translate to caregiving willingness and vice versa. However, this is currently being explored as part of a large systematic review and a multinational empirical study (see: www.entwine-itn.eu).

### Motivations and willingness to provide care as expressions of intention

It is essential to recognise that both motivations and willingness to care are expressions of intentions and do not inevitably lead to actual behaviours. Although socio-cognitive models of human behaviour (such as the Theory of Planned Behaviour; Ajzen, 2011) posit that an individual’s intention, which may be operationalised as willingness arising from underlying motivations, is the immediate precursor/predictor of engagement in a behaviour (Ajzen, 2011; Brayley et al., 2015; Reuveni & Werner, 2015), the disconnect between attitudes and behaviour remains and many factors have been studied which appear to begin to fill this ‘gap’ (e.g. Sniehotta, Presseau, & Araújo-Soares, 2014). The issue of the impact of intention-based caregiver motivations and willingness to care on the actual behaviour remains unexplored in the caregiving research: How do caregiving motivations explain carer’s intention to care and does intention differentially relate to behaviour when the underlying motivations are extrinsic versus intrinsic, for example? Are caregiving intentions transferred into action at all and if so in what circumstances? These are just a couple of questions that require investigation.

Where the Theory of Planned Behaviour has been utilised in studies of willingness and motivations to provide care (e.g. Goldberg-Looney et al., 2017; Katz, Gur-Yaish, & Lowenstein, 2010), it has been assumed that personal attitudes and subjective norms (of caregiving behaviours) influence behavioural intentions, but not the reverse. Evidence on the relationship of motivations (operationalised mainly as filial norms) to actual caregiving behaviour appears ambiguous: with some evidence that filial motivations play a role in the amount of help provided (e.g. Ikkink, Van Tilburg, & Knipscheer, 1999; Silverstein, Gans, & Yang, 2006), although others have not confirmed this relationship (e.g. Eggebeen & Davey, 1998). The inconsistency in findings might reflect cultural variations within the studies but it may also relate to the limited predictive validity of the Theory of Planned Behaviour which has been shown to be most predictive amongst the young, fit and affluent when predicting self-reported behaviour over a short term (Sniehotta et al., 2014). The exclusive focus on rational reasoning expounded by such models, excluding unconscious influences on behaviour as well as the role of emotions and social desirability, may, in our opinion, limit the usefulness of the Theory of Planned Behaviour in the caregiving context, especially given the complex conceptualisations of motivations to provide care. Moreover, beliefs are often found to predict behaviour over and above intentions (Sniehotta et al., 2014), possibly positioning the role of the intention-oriented research among a wider spectrum of caregiving investigation of motivations to care. Neither motivations nor willingness to provide care may lead to actual behaviours given the important contextual factors (e.g. deterioration in their care recipient’s health condition, caregiver’s own health, flexibility to accomodate caregiving with existing employment commitments) that may constrain the influence of intention on the behaviour. Given this, it is important to reiterate the importance of studies which have addressed these constructs in relation to outcomes of caregiving, as presented below.

### Influences of motivations and willingness to provide care on caregiver outcomes

In this section we present a brief summary of the empirical evidence pertaining to the impact of motivations and willingness to provide care on caregiver outcomes. Although research on this subject is limited, findings highlight the challenges in operationalising the two constructs and their actual impact on caregiver behaviour.

Willingness to provide care has been positively related to caregiver burden and stress, perhaps due to an over-investment in the care role (Burridge et al., 2007; Gupta, Rowe, & Pillai, 2009; Zhan, 2006). It has also been shown that norms of perceived obligation towards family members, familism/filial values and ethnicity have a predictive value for the actual exchange of informal care (Dykstra & Fokkema, 2012; Goldberg-Looney et al., 2017; Ikkink et al., 1999; Morrison & Williams, 2020; Parveen et al., 2011, 2013, 2014; Silverstein et al., 2006).

Reluctance to care, which can be considered the converse of willingness to care, has been shown to have negative repercussions upon the caregiver, care recipient, and other family members’ quality of life. Many caregivers who self-report feeling reluctant, even obligated, experience greater depression and greater anger than those expressing willingness, and provide a lower quality of care which can include potentially harmful psychological and physical behaviour towards the care recipient, and a deterioration in the carer–recipient relationship (Burridge et al., 2007; MacNeil et al., 2010; Raveis, Karus, & Siegel, 1998). Although willingness to provide care may mitigate the negative consequences for carers and care recipients, as indicated before, it also comes at a cost. Willingness can be related to burden and stress, possibly due to an over-investment in the care role (Burridge et al., 2007), described in a qualitative study conducted by Morrison and Williams (2020) as ‘consuming the role’.

Generally, it remains unclear whether willingness to provide care has positive or negative outcomes and for which sub-sample of carers (e.g. dementia carers, spousal carers, etc.). This conclusion may reflect the temporal, dynamic nature of the notion, as discussed before (also in relation to the ‘caregiving journey’) supporting our understanding of willingness to provide informal care as a process. Moreover, as suggested by Burridge et al. (2007), there might be an element of taboo surrounding the topic of caregiving willingness to provide care as expressing reluctance to care may be socially undesirable.

The previously considered temporal orientation of motivations to provide care, i.e. changing caregiving motivations (initiation and continuation motives) may also shed more light on the inconsistent findings regarding how motivations impact caregiver outcomes. Generally, studies have shown that intrinsic motives lead to more positive caregiver outcomes than extrinsic motives, with differences noted in coping strategies, emotional distress, feelings of burden, quality of care, caregiver satisfaction and stress (e.g. Burridge et al., 2007; Carruth, 1996; Donorfio & Kellett, 2006; Dumit, Abboud, Massouh, & Magilvy, 2015; Knight & Sayegh, 2010; Lyonette & Yardley, 2003; Parveen et al., 2011; Romero-Moreno, Gallego-Alberto, Márquez-González, & Losada, 2017). However, we should not easily conclude an advantage of one or other type of motivation as research addressing extrinsic motivation, based on, as described previously, incentives and outcome expectancies has also found these motives to be associated with positive caregiver outcomes (Burridge et al., 2007; Qiu, Sit, & Koo, 2018; Rohr & Lang, 2016; Tang, Li, & Liao, 2007; Vroman & Morency, 2011; Youn, Knight, Jeong, & Benton, 1999). This inconsistency in results supports our proposed consideration of an interactive juxtaposition of extrinsic and intrinsic motivations on caregiver outcomes.

Care recipient’s nursing home placement, although generally seen as inappropriate by caregivers (e.g. Kong, Deatrick, & Evans, 2010) due to caregivers’ beliefs (such as for example negative beliefs and perceptions of nursing homes, their familial obligation), further illustrates how motivations may impact on caregiving behaviour. For instance, Ho, Friedland, Rappolt, and Noh (2003) reported qualitative findings that Chinese dementia carers made applications to nursing homes against their previously stated motivations of providing care due to cultural and personal values. This suggests that caregiving motivations and willingness are dynamic in nature as they can be renegotiated and restructured depending on the context – as seen in the aforementioned example (Ho et al., 2003) or other similar instances (Han, Choi, Kim, Lee, & Kim, 2008; Hsueh, Hu, & Clarke-Ekong, 2008; Kim, 2009; Kodwo-Nyameazea & Nguyen, 2008).

## Conclusions

As discussed above there is a need for prospective longitudinal designs in research examining caregiving motivations and willingness to provide care as these are dynamic in nature. We have proposed that different motivational factors may be present at different stages of a ‘caregiving career’ or ‘caregiver journey’ subject to changes in caregiving needs or as carer experience grows (Alonso, Kitko, & Hupcey, 2018; Boeije, Duijnstee, & Grypdonck, 2003; Browne Sehy, 1998; Foster, 2012; Ho et al., 2003; Hsu & Shyu, 2003; Kong et al., 2010; Lin, Macmillan, & Brown, 2012; Morrison & Williams, 2020; Opie, 1994; Parveen et al., 2011; Quinn, 2009; Sasat, 1998; Stajduhar et al., 2008; Williams et al., 2014). Furthermore, as motivations can change over time, we also propose that initiation motives be distinguished from continuation motives. In addition, the factors influencing motivations to provide care over time should be explored and in doing so it will be critical to address the contextual factors around the provision and receipt of caregiving such as family structure and the quality of relationships, working arrangements, geographical proximity, and the personal and financial means to provide care.

We have attempted to highlight that motivations and willingness to provide care remain inappropriately and inconsistently conceptualised and defined as well as understudied in informal care research. As shown, motivations are multifaceted and multiply determined (Baker et al., 1988; Kanfer et al., 2008; Morrison & Williams, 2020; Wallroth, 2016) and contrary to early descriptions of intrinsic and extrinsic motivations as antagonistic we provide evidence of their coexistence (e.g. Morrison & Williams, 2020). We suggest that the current model of extrinsic and intrinsic motivations is oversimplified and has omitted consideration of the diversity of caregiver motives, and indeed of other motivational models discerned in the context of the empirical research (Abell, 2001; Adams et al., 1976; Barber, 2010; Bateson, 1991; Blieszner & Shifflet, 1989; Clark & Mills, 2011; Frank, 2002; Greenberg, 1990; Homans, 1961; Humphrey, 1997; Johnson et al., 1999; Le et al., 2018; Lind & Tyler, 1988; Ryan & Deci, 2000; Vallerand, 1997). The theoretical approaches and the methods of assessments pertaining to caregiving motivations should therefore go beyond the basic dichotomy of intrinsic and extrinsic motives, i.e. more complex theoretical models recognising the diversity and different levels of motivations should be developed and applied in future research in order to reduce the arising inconsistencies in current empirical research.

Given the above demonstrated significance of these constructs and theories, we would suggest that future research considers the following: caregiving motives; reasons for providing informal care; justifications for caregiving; caregiving obligations; filial expectancy; filial/familial values; filial piety; reciprocity; perceived or actual choice to provide care; caregiving involvement; commitment to support; caring drive; and caregiving duty. We have also highlighted the lack of clear definition and consistent operationalisation of these concepts as well as their multifaceted and diverse nature in the field of informal care. It will be important to address these limitations in subsequent caregiving research if findings are to be comparable and theoretical and methodological advances are to be achieved.

Although we have shown that there are similarities between the constructs of motivations and those of willingness to provide care, we argue that willingness to perform care is a more behavioural attitude with current (actual) and future (hypothetical) orientations which may themselves vary depending on the orientations (dimensions) of a person’s motivations to provide care. Motivations and willingness may, however, not inevitably lead to behaviour and as is seen in many studies of human behaviour, when understood as expressions of intention, motivation and willingness may offer insufficient explanations of whether care tasks are performed or not (Eggebeen & Davey, 1998; Sniehotta et al., 2014). This is due to other contextual factors (e.g. caregiving skills, cultural norms, geographical proximity) about which more needs to be known. Developing and operationalising coherent definitions in robust and ecologically valid research is necessary before a synthesis of findings can follow. This is needed if appropriately timed and tailored interventions, which seek to optimise motivations and willingness to care and be translated into supportive care provision, are to follow.

Finally, the synthesis presented here holds relevance for support services and health and social care practitioners. Increasing understanding of the differences between motivations and willingness to provide care, and in the factors that influence these, helps to identify potential targets for future intervention studies, with the goal of sustaining caregiving motivations and willingness for caring. We need support sensitive to caregivers’ unique circumstances, which distinguishes between initial motives and continuation motives, between ability and willingness to provide care and which recognises variations in such factors over time. We would caution against making assumptions about caregivers’ motivations and willingness to care.

Given that the availability and continuity of informal caregiving is a global requirement (if stretched formal services are to be supported), this paper usefully points to a need for research which addresses the internal, individual, context-based experience whilst also considering moral and ethical aspects of caregiving motivations (Goldsteen, 2008). For example, what factors (personal, social, moral) influence decision-making processes leading to assuming or relinquishing the caring responsibility, decision processes regarding a nursing home placement, dilemmas relating to the negotiation of a care recipient’s autonomy (such as for instance managing their finances), the extent of the caregiver’s responsibility/obligation, or the question of who should coordinate the process of care? Motivations and willingness to provide care should also be considered within the socio-cultural context in which they operate, and better understanding of these factors and their influence have relevance for health and social care practice or policy. As indicated before, the distinction between ability and willingness to provide care is important here: any caregiver assessment should leave space to explore caregivers’ diverse motivations and willingness for caring as well as their unique circumstances in a timely way once their ability to care has been confirmed. Many of the questions raised in this commentary paper remain to be answered, and answers to these questions will bear relevance for research, policy and practice, and therefore supportive and effective care provision.
